# Understanding market functionality and trading success

**DOI:** 10.1371/journal.pone.0219606

**Published:** 2019-08-21

**Authors:** James Schmitz, David Rothschild

**Affiliations:** 1 Tisch School of Arts/ITP, New York University, New York City, NY, United States of America; 2 Microsoft Research, Microsoft, New York City, NY, United States of America; National Institute of Public Finance and Policy, INDIA

## Abstract

We examine individual-level trading data from several markets in the PredictIt exchange to determine what strategies correlate with financial success. PredictIt provides many markets with futures contracts linked to political issues, ranging from ongoing policy outcomes to political elections. High fees along with restrictions blocking automatic trading and constraining a one-to-one match between people and accounts, combine to severely limit the upside to investment returns over the fixed costs: this ensures that traders are all retail investors. We have the individual-level data from two markets: Democratic and Republican Iowa Caucuses in 2016. This data includes all orders and trades from every trader across the markets. We are able to fully reconstruct market activity and study trader behavior both within and between markets. We show that understanding how markets and trades works is more important to financial success than proxies for (1) confidence or funding (2) information or objectivity in trading. The work should be a call-to-action in favor of simplifying markets and trading for any exchange with retail investors, and for more research into effects of differential trading efficiency in all financial markets.

## Introduction

We examine individual-level trading data from several markets to determine what strategies correlate with financial success. Specifically, we examine trading data from PredictIt, which is a prediction market exchange that provides many markets linked to political issues, ranging from ongoing policy outcomes to political elections. The exchange was created before the 2016 election and quickly gained a robust set of users. The exchange caps traders at $850 in exposure for any contract per Commodity Futures Trading Commission guidelines. The exchange vigorously identifies each user to ensure they have only one account, and blocks automated trading. Further, all contracts are capped at 5,000 traders, but that was not an issue for any contracts in the markets we examine. In addition, the exchange charges users 5% of their all of their money in the exchange (including both their investment and profits) when withdrawing money. Further, there is a 10% charge on all profitable trades. These restrictions certainly make this exchange unrepresentative of many other futures exchanges. It is impossible to justify institutional investors covering both the fixed cost of entering the exchange, and any given market, with these low limits and high fees. But this allows us to test something interesting: what can we learn about trading patterns in markets filled completely with retail investors?

We have the individual-level data from two markets: the Democratic and Republican Iowa Caucuses preceding the 2016 presidential election. This data includes all orders and trades from every trader across the markets. We are able to fully reconstruct market activity and study trader behavior both within and between markets. This task was both non-trivial and interesting. Exchanges save data in order to make sure they are functioning properly, but that is not necessarily the same data one would need to adequately research individual-level trading patterns or the aggregate prices that emerge.

There is a great deal of research on prediction markets doing a good job in aggregating data into prices that somewhat accurately reflect the probability of upcoming events [1, 2]. Like all markets, the efficiency of the price depends on institutional investors acting as marginal investors, driving the prices towards the efficient price. There is much less research on the price efficiency of markets that disincentivize institutional investors.

There are a selection of papers that examine the trading inefficiencies of prediction market traders in more open exchanges. [3] examines the order books in both Intrade and Betfair in 2012 and shows how traders prefer to buy over sell, inefficiently pushing the sum of all bids in a set of mutually exclusive contracts (where one will be worth $1 at the end of an event) much closer to $1 than the sum of all asks. This potentially would have made it more valuable for traders looking to go long on a particular outcome to sell all other outcomes, but the authors did not have the trading data to examine this. [4] has access to individual level trading data on Intrade and shows how most traders are exclusively long in one direction. But, both of these exchanges are not capped, and the papers do not address the competitive impact of these inefficiencies within the markets, between the traders.

Several papers have taken a detailed look the Iowa Electronic Market (IEM), which is interesting because it is the only other major exchange, besides PredictIt, that constrains amount of investment. That exchange limits traders to a $500 initial investment in the exchange, which would make institutional investing difficult as well. [5] investigates manipulation in the IEM in 2008. A key result is that without wealth disparities it is hard for anyone to manipulate or drive the market. Another key paper [6] shows the inefficiencies of traders from the perspective of a single large trader in the exchange. The research team at Iowa has also written many papers about their exchange [7].

Turning to the more general finance literature, there are competing theories on why trades happen. First, various differences in risk or liquidity makes it so people with more money and patience can profit from other traders. In this model, funding is the key to better returns [8–10]. Second, there can be heterogeneous priors or models. Some people just interpret the world differently and money is made by the traders with better interpretation of the information [11, 12]. Neither sets of theories allow for differences in traders’ understanding of the exchange, market, and specific assets being traded.

There is a nascent thread of finance literature that exploits individual-level trading data on traditional assets to help tease out trading patterns. [13] explores prospect theory in set of traders from an unnamed online financial trading platform. [14] uses individual-level trading data to parse out algorithmic trading patterns. This paper adds to a growing interest in large-scale individual-level trading data to understand market efficiency and trading strategy.

This paper also speaks to finance literature on the inefficiencies coming from high-frequency trading [15]. After describing the problem, we propose that exchanges recognize uneven returns that are not related to trader knowledge or liquidity, but are germane to the running of the exchange itself.

Overall, this paper is unique in that we have individual-level data, and are constrained to retail investors, who demonstrate a meaningful lack of understanding of the assets being traded. We use a proxy for information or objectivity to see if traders with better models of the data make more money than non-information traders. We use a proxy for funding or confidence to see if bigger, more funded traders make more money than smaller, less funded traders. This follows the two major theories of trading. Finally, we examine the efficiency of the traders in how they make trades, a proxy for how well they understand the actual workings of trading, and examine if that is better indicator of success than the other two, more traditional indicators. This is a comparison we have never seen examined in the literature.

These three variables are correlated: if a trader has one of these attributes, that trader is more likely to have any other attributes. It is impossible for us to fully disentangle causality that from our existing data. We are not observing a trader with two of the variables, handing him or her the third variable, and making any claims about causation. A trader who understands the working of markets well is also more likely to have money to invest, and means to model information. But we have enough traders with two of the variables, and by observing variation in the third, get a solid understanding of what correlates with better returns on investments.

We show that understanding how markets and trades works is more important in success than either proxies for objective models of information or funding. Within classes of traders who possess or do not possess a given trait, there is nothing more indicative to success in the markets than having the ability to spot the most efficient way to make a given wager. Objective models for information are also correlated with positive returns, but more funding does not necessarily correlate with higher returns. These results should be a call-to-action for simplifying trading for exchanges or contracts aimed at retail investors.

## Data

PredictIt provided us with historical data for the 2016 Democratic and Republican Iowa Caucus elections. The data contained information on new orders, order modifications, and executed trades with unique trader IDs that allowed us to identify all of each trader’s activity while maintaining anonymity. Using this data we could analyze each trader’s profitability and trading style.

In addition to the data provided to us by PredictIt, we had top-of-book quote data collected every five minutes using PredictIt’s publicly available API. This data wasn’t used directly in this paper but was used to help evaluate the accuracy of some of our data cleaning efforts.

### Basic market structure

It is important to understand the basic structure of the PredictIt markets and contracts. The markets included a finite set of mutually exclusive outcomes with one contract representing each candidate in the actual Iowa Caucus election. In order to wager in favor of a candidate, a trader has two choices: buy Yes contracts for that candidate or buy No contracts for any other candidate. Buying No contracts also provides the trader with a profitable outcome if any other candidates wins (not just the one candidate for which they would have bought a Yes contract for).

Understanding the difference between Yes contracts and No contracts and the decision-making process between trading one or the other is central to the thesis of this paper.

At market termination holders of each Yes and No contract will receive $1 if the contract definition is satisfied and $0 if it is not. In the Iowa Caucus markets, a Yes contract would be worth $1 if the named candidate actually won the caucus and $0 otherwise. For a No contract it will be worth $0 if the named candidate wins and $1 if the candidate loses.

Traders are presented with a visible order book showing the demand to buy or sell each contract. All orders are priced in penny increments between $0.01 and $0.99.

The PredictIt exchange charges traders fees based on their trading activity. PredictIt collects a 10% fee on individual positions that are sold at a profit and no fee on positions that are sold at a loss. The exchange uses a first in, first out accounting model to identify which shares are sold and to calculate their fee. Before fees these markets are essentially zero sum games with unprofitable traders losing money to profitable traders.

### Data cleaning

We were not able to reconstruct the full order book because a portion of the order cancellation events were not recorded. We investigated with help from the PredictIt team and learned that PredictIt had a nightly process that removed unfeasible orders when traders no longer had the capital to cover an order and orders that would potentially put traders over the $850 contract limit. For much of our dataset these unfeasible cancellations were not recorded. The missing information could not be recreated, and its absence undermined our ability to reconstruct the order book.

Since the order information was flawed we developed an alternative approach to reconstruct the top-of-book prices using only the executed trade data. We know the times and prices of all of the executions and we also know the times the orders were sent. For any matching trade we can figure out which order provided liquidity because it will be the order that was placed in the order book first. Using the prices of the orders that provided liquidity we can infer the top-of-book price on one side of the order book at the time of each execution.

Using these inferred top-of-book prices we can make an assumption that these prices remain at the top-of-book until the next provide execution gives us new information about the top-of-book price. This, combined with the knowledge that the bid-ask spread must always be one penny or more, allows us to reconstruct a complete and usable set of top-of-book prices for all contracts over the life of the market.

An important question is how well this procedure approximates the top-of-book prices that would be derived from an ideal dataset. To evaluate this we can compare these prices with the five minute sampled data we collected from the PredictIt API. We performed this comparison and found that it does provide a good reconstruction of the top-of-book prices. It performs best during periods of peak trading activity, which is ideal for this research project.

### Margin linking

There was a significant change to the mechanics of the PredictIt markets that took place towards the end of 2015, and that change is the introduction of something called “margin linking.” To understand what this means, first consider the PredictIt trading system applying basic accounting by deducting funds from a trader’s account whenever the trader make a trade to cover a possible loss in the contract. Now consider a trader that buys multiple No contracts that are linked together in a market such as the Iowa Caucuses that allows only one candidate to be the winner. In this case it simply isn’t possible for all of the trader’s No contracts to lose money; at most only one of the No contracts can lose money. Therefore if the PredictIt trading system were to deduct the total cost of each of the No contracts the system would be deducting more money from the trader’s account than the trader could lose in any possible market outcome.

The introduction of margin linking changed the PredictIt exchange so that it considered the maximum possible loss a trader could suffer across multiple positions in contracts in a linked market. Before margin linking the PredictIt exchange would deduct funds from a trader’s account without considering linked outcomes; after margin linking the exchange considered linked outcomes and deducted only the maximum possible loss the trader could actually suffer. This new feature was rolled out to markets gradually over several weeks and resulted in additional tradable funds being added to some traders’ accounts. Traders were informed of this change several weeks in advance and market prices started to adjust in advance of the margin linking rollout.

**Fig 1:**
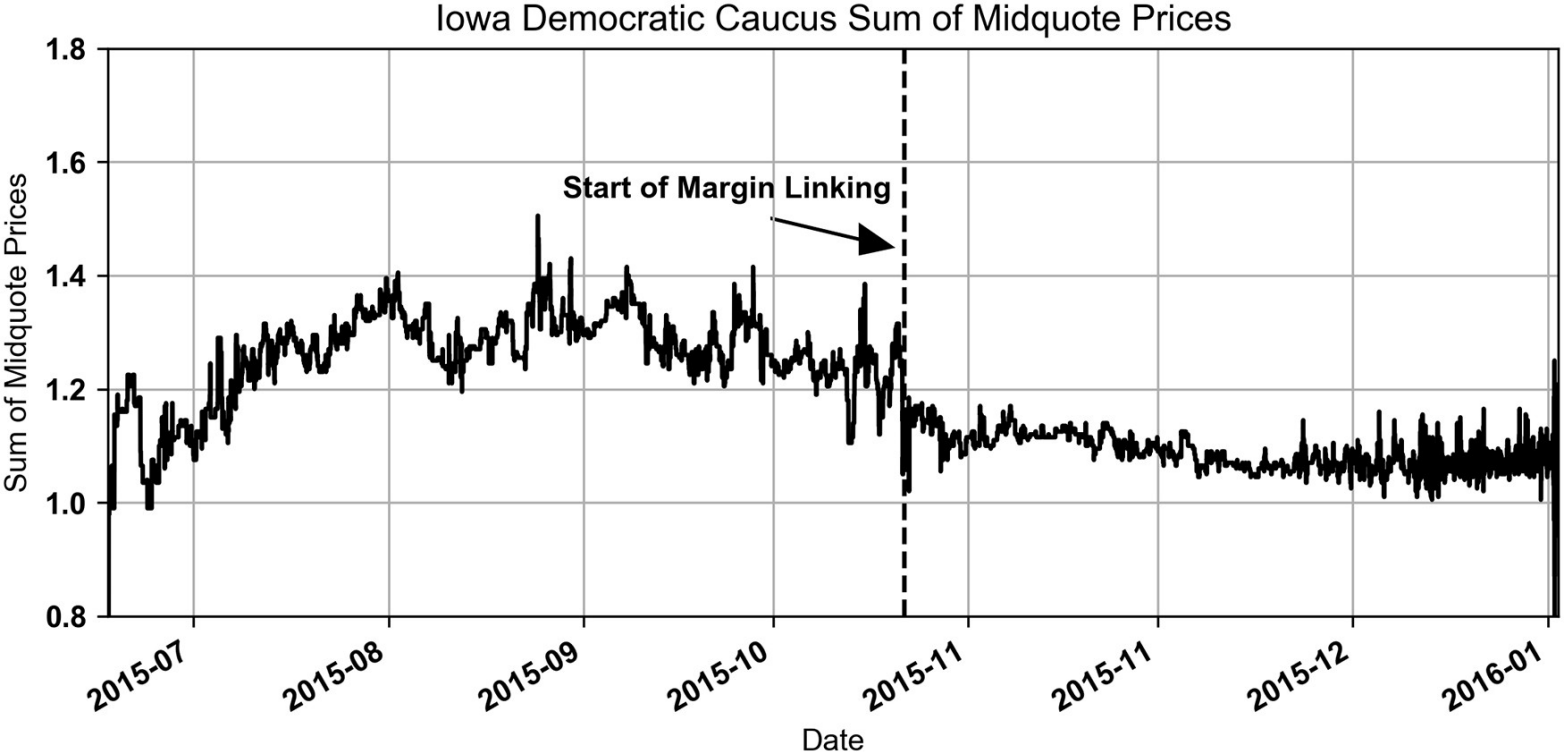
Iowa Republican Caucus sum of midquote prices.

**Fig 2:**
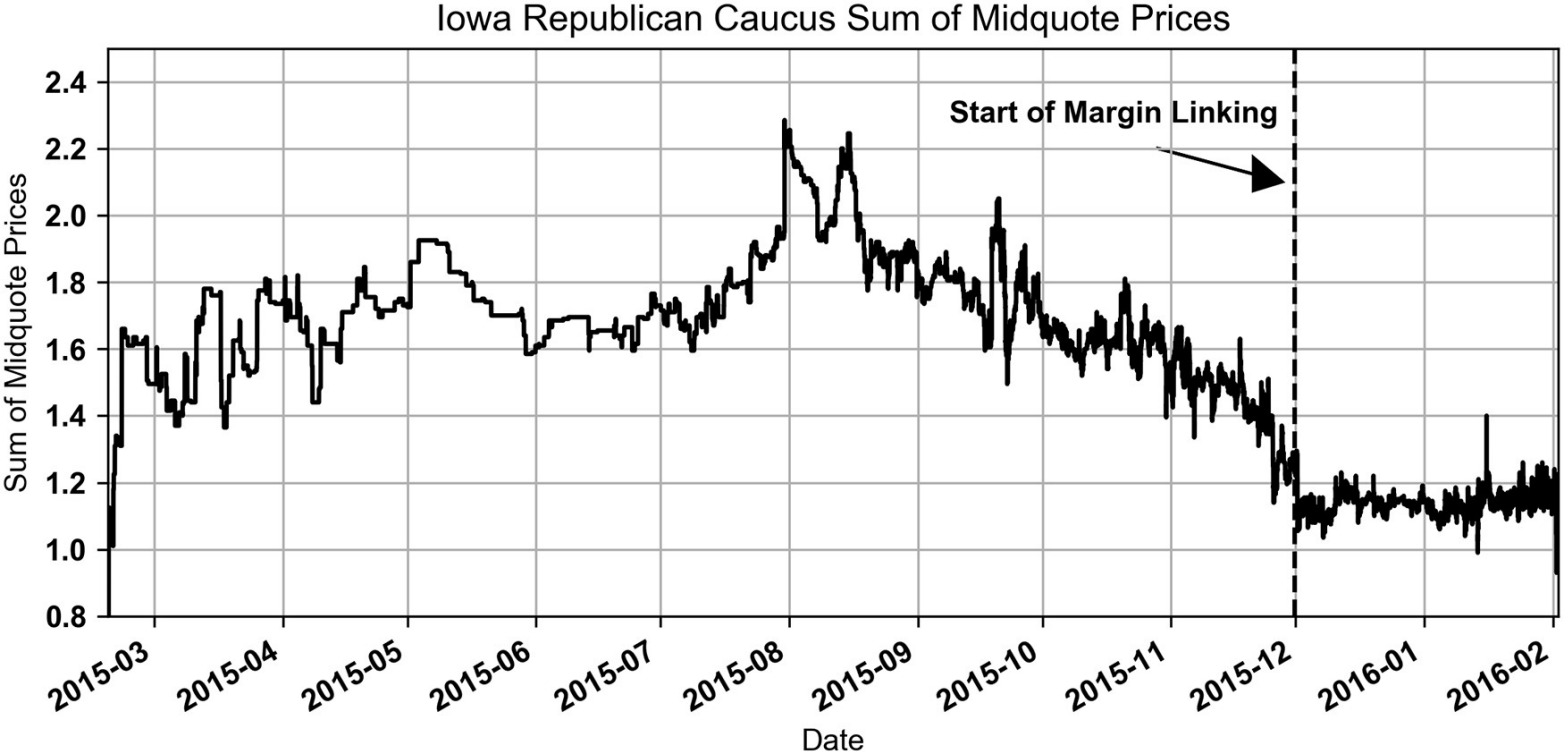
Iowa Democratic Caucus sum of midquote prices.

The impact on prices can be seen by looking at the sum of the midquotes (average of bid and ask) for all of the contracts available in a market, shown in Figs 1 and 2. Before margin linking went into effect in the Republican Caucus market, the sum of the midquote prices peaked over $2. Consider that in a more well functioning prediction market the sum of the midquote prices would be close to $1. After margin linking was added on December 1, 2015, the sum decreased to the $1.10 to $1.20 range. Margin linking went into effect in the Democratic Caucus market on October 22, 2015 and had a similar impact on prices. In both markets traders were able to move prices down using the additional funds that were added to their accounts. This had the effect of making prices more fair for traders buying Yes contracts.

Although margin linking had a large impact on market structure it would be impossible and incorrect to only include the post-margin linking data in this study. However, only 5% of the Democratic shares and 8% of the Republican shares traded during the pre-margin linking period, making it impossible to exploit the change to test the efficiency change of the traders.

### General market activity

We can explore our cleaned market data to gain an understanding of general market activity.

The Republican and Democratic Iowa Caucus markets started in February and June of 2015, respectively. Both markets concluded on February 2nd, 2016, with the bulk of the trading activity taking place during the days and weeks leading up to the actual election day.

**Fig 3:**
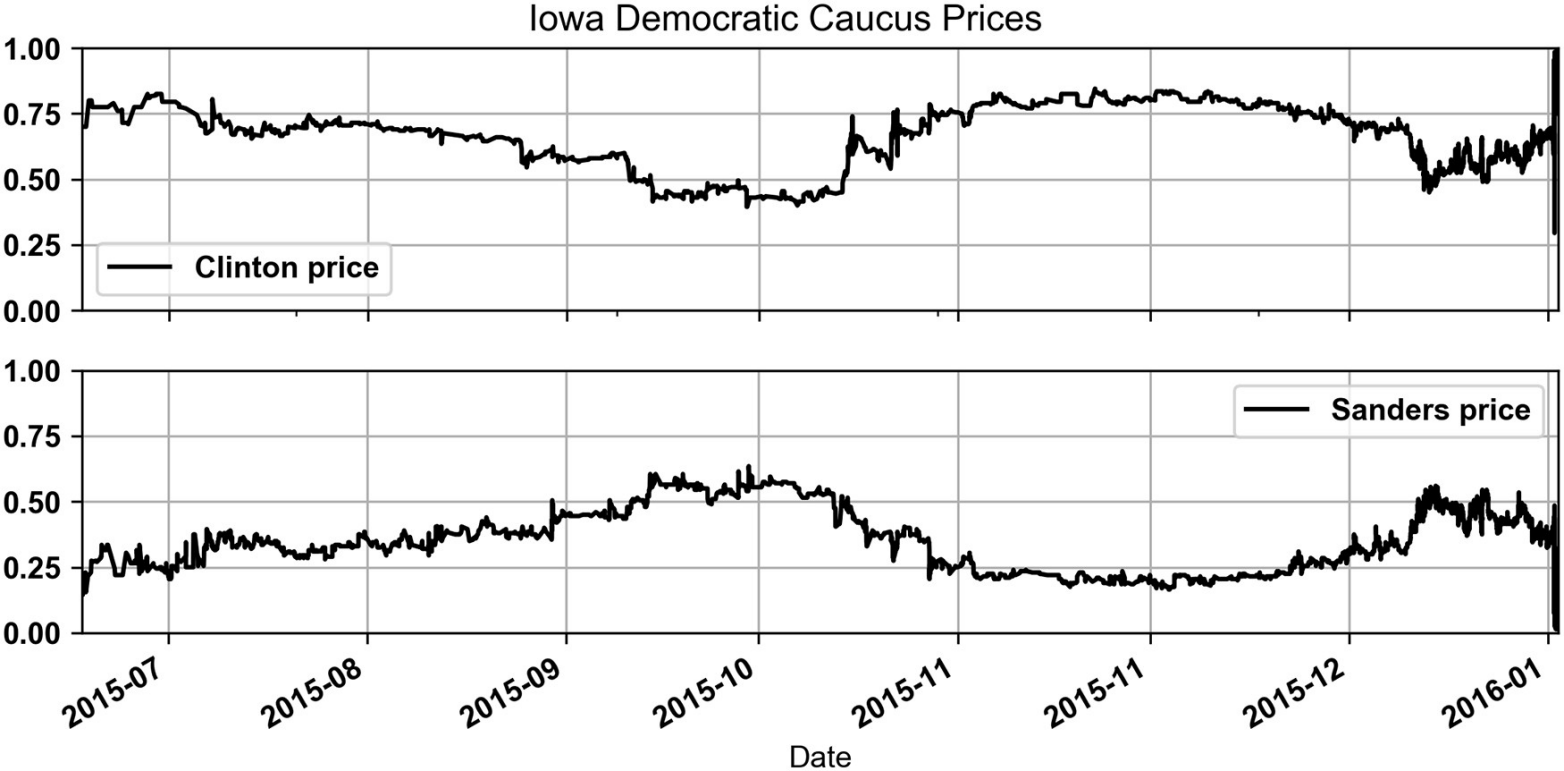
Iowa Democratic Caucus prices for Clinton and Sanders.

As shown in Table 1, most of the trading activity in the Democratic Caucus was for candidates Sanders and Clinton. For most of the duration of this market the price of the Clinton security suggested that she was the likely winner; ultimately she did in fact win the Iowa Democratic Caucus. Fig 3 shows the security midquote prices for both candidates with the price of the Clinton security above $0.50 most of the time.

**Table 1:** Iowa Democratic Caucus total trading activity by candidate.

	Shares Traded	Notional Traded ($)
Sanders	1,327,990	659,721.16
Clinton	1,223,458	568,022.08
O’Malley	44,048	23,622.64
Biden	41,566	18,150.80
Webb	5,328	2,288.70
Chafee	8	5.96

**Fig 4:**
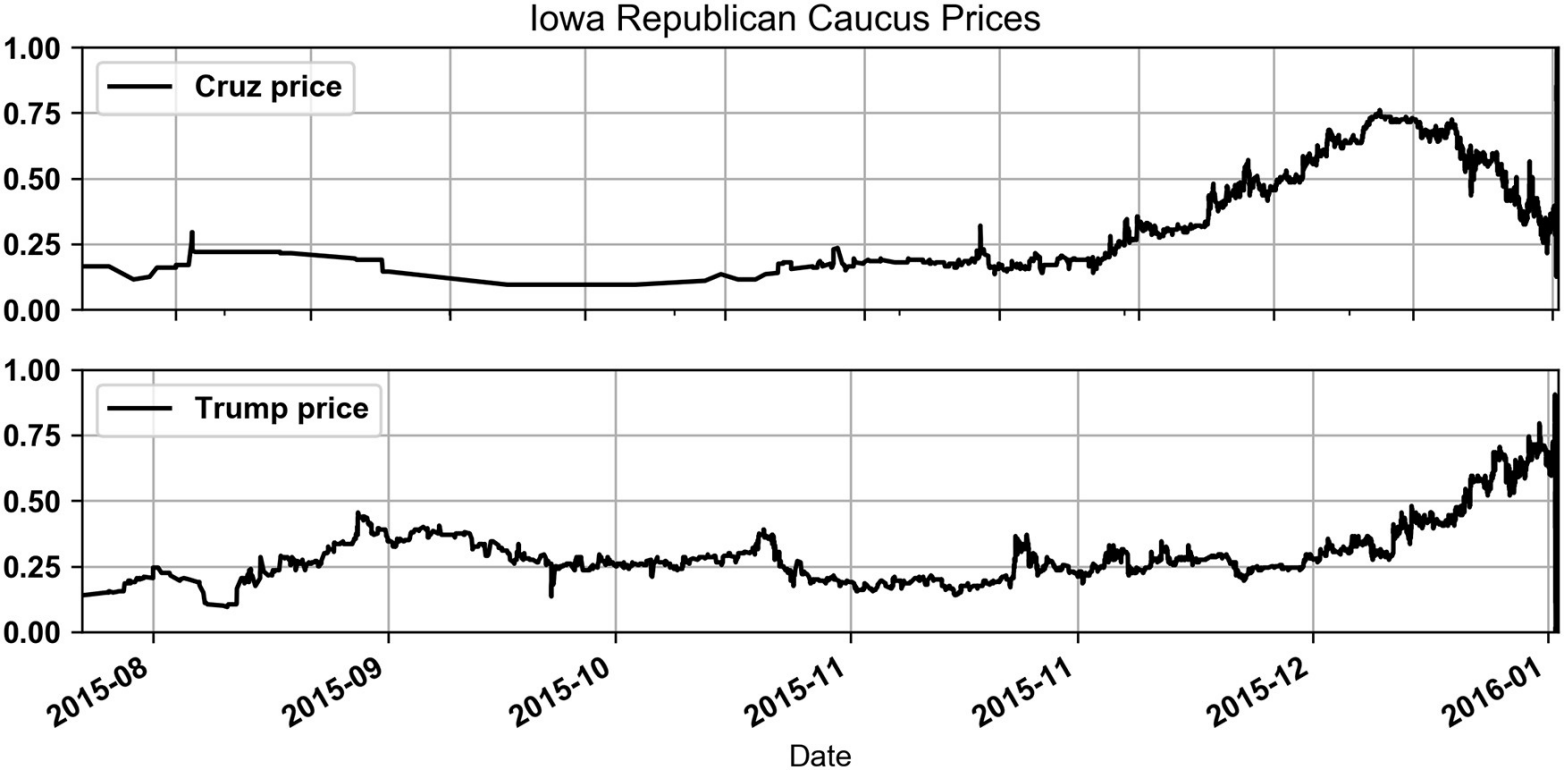
Iowa Republican Caucus prices for Trump and Cruz.

In the Republican Caucus there were more candidates and there was more uncertainty about who would win. Table 2 shows that candidates Trump and Cruz had the highest trading volumes but other candidates such as Paul, Rubio and Carson also saw significant trading volume. In the months leading up to market termination the prices shown in Fig 4 suggest that first Cruz and later Trump was the likely winner. Cruz ultimately won the election but prices suggested that Trump was still expected to win up until a few hours before the market concluded. The higher uncertainty about the outcome of this market impacted trader profitability and the results of this paper.

**Table 2:** Iowa Republican Caucus trading activity by candidate.

	Shares Traded	Notional Traded ($)
Trump	1,504,976	755,815.48
Cruz	1,315,862	663,317.44
Paul	834,450	421,771.84
Rubio	957,920	411,231.44
Carson	685,058	315,323.40
Huckabee	182,680	76,436.52
Bush	168,766	67,130.60
Christie	101,940	49,189.62
Santorum	78,572	39,330.02
Fiorina	69,442	32,976.98
Kasich	59,502	30,317.42
Jindal	35,196	16,366.44
Walker	17,804	10,900.46
Perry	4,302	2,182.70

Millions of dollars and shares were traded during the lifetime of both markets. Table 3 shows that the Republican market attracted more than twice the trading activity than the Democratic market and that PredictIt collected more than twice the fees.

**Table 3:** Summary of total market activity in Iowa Caucus markets.

	Shares Traded	Notional Traded ($)	PredictIt Fees ($)
Democrat	2,642,398	1,271,811.34	11,718.86
Republican	6,016,470	2,892,290.36	23,096.63

The majority of the trading activity took place right before market termination. Table 4 shows the daily trading volume in shares for select dates, along with the percentage of cumulative trading volume by date. It is clear from this table that in both markets more than half of the volume took place in the last few days before market termination.

**Table 4:** Market activity by date. The Volume column shows the total shares traded on each day and the Cum. Volume % column shows the percent of total volume that took place on or before that date.

	Democratic		Republican	
	Volume	Cum. Volume (%)	Volume	Cum. Volume (%)
2015-07-01	736	0.1711	430	0.1914
2015-09-01	512	2.32	1,894	0.9952
2016-01-01	8,312	12.75	20,496	23.76
2016-01-13	14,494	18.00	62,560	31.06
2016-01-20	21,626	23.97	48,224	36.37
2016-01-27	44,000	35.55	92,142	46.24
2016-01-28	33,504	36.82	142,114	48.60
2016-01-29	66,666	39.34	200,356	51.93
2016-01-30	75,296	42.19	278,388	56.56
2016-01-31	117,420	46.64	282,128	61.25
2016-02-01	392,516	61.49	753,354	73.77
2016-02-02	1,017,550	100.00	1,578,254	100.00

### Trader behavior summary

After all of the data was cleaned we were able to analyze trader behavior and calculate profitability metrics.

Table 5 provides a topline summary of trader activity in the two markets. There were 3,750 and 4,452 traders who executed at least one share in the Democrat and Republican markets, respectively. With 2,042 traders in both markets, there are 6,160 unique traders (i.e., 2,042 in both markets, 1,708 in just Democratic market, and 2,410 in just Republican market). Note that “Average (pre-fee) Profit” is zero by definition because before fees this market is a zero sum game. After fees the average trader suffered a small loss, with the range of profits or losses within a few thousand dollars. Also note that “Money Risked” is the total amount of money that was exposed in the market (only counting the same money once) and contributed to the zero sum game by each trader at any time during the life of the market. The Average and Median “Holding Time” statistics are weighted by shares traded. Holding times are skewed, with 66.8% and 72.3% of Democratic and Republican traders holding positions until market termination.

**Table 5:** Summary of trader activity in the Iowa Caucus markets.

	Democrat	Republican	Combined
Traders	3,750	4,452	6,160
Orders Sent	33,959	62,551	96,510
Money Risked (cumulative exposure, $)	417,251.70	544,205.11	961,456.80
Average Money Risked (cumulative exposure, $)	111.27	122.24	156.08
Average Holding Time (days)	13.40	16.26	15.39
Median Holding Time (days)	1.68	4.73	3.73
Average (pre-fee) Profit ($)	0.00	0.00	0.00
Average (net-fee) Profit ($)	-3.13	-5.19	-5.65
Min (net-fee) Profit ($)	-1,699.58	-1,701.32	-2,429.86
Max (net-fee) Profit ($)	1,187.54	2,882.00	3,101.20

We classified traders by three key metrics: their maximum market exposure (proxy for funding or confidence), willingness to open and close contracts multiple times (proxy for information or objectivity), and if they made efficient trades (traded in the most cost-effective way to make a given wager).

The size of a trader’s exposure says something about how serious they are, confident they are, and how well funded they are. Larger traders are more likely to be risk neutral and lack liquidity constraints, and they they are less vulnerable than small traders to lose money on trades due to monetary objectives beyond maximizing future expected wealth. We simply characterize traders as “small” or “large.” We use $50 in exposed risk as the boundary between the two groups, but this paper’s results are robust to adjusting that value.

Another way to classify traders is whether or not they actively traded into and out of positions on a single contract or if they followed a passive buy-and-hold approach. This is a good proxy for an information trader who is trusting his or her trading skills rather than trading on their personal political views or naive interpretation of the available information. We use a simple threshold: did the trader ever close a position and re-open it on a single contract? This includes traders that switched the direction (from Yes contracts to No contracts, or vice-versa) of their position and traders that closed and re-opened positions in the same direction. We classify these traders as active traders (as opposed to inactive traders) because they are more frequently adjusting their position. This is a binary classification, so there is no way to test the robustness, but we believe that any trader that ever changes her position is, at minimum, not a fully partisan trader and is acting on information that leads her to believe that her subjective probability of an outcome is far enough from the current price to actually switch direction. Further, a fully rational trader, with no opportunity costs, is unlikely to be long in one direction for the entire length of a contest with high information flows. Of course, some traders will rationally calculate is is not worth their time to trade beyond their initial informed trade, even if new information indicates that their position is no longer optimal. Thus, our classification is a precise definition insofar as most of the people we classify as active are informed, but there will be some informed traders in the inactive group.

A trader is classified as efficient if he or she were able to identify the most cost-effective way to make a given wager with at least 75% of their trades being efficient. As we will show later in this paper, the availability of Yes and No contracts in these markets provides traders with two trading options because Yes contracts can be replicated with No contracts and No contracts can be replicated with Yes contracts. The two options were rarely equivalent as one was frequently more cost-effective than the other. An efficient trader is one who is able to make the most cost effective choices. This is explained in detail in the Evaluating Trader Efficiency section. This paper’s results are robust to adjusting the 75% value.

Table 6 shows that in both markets most traders are small and inactive. With activity being a binary variable, it is impossible for us to consider more comparably sized groups (we will later show that the vast majority of traders are also inefficient). Trading theory assumes that small and inactive traders would lose money to large and active traders. As large active traders, without liquidity constraints and using optimized models, would take money from small and inactive traders who are potentially liquidity constrained (due to non-expected return maximizing trades) and using inefficient or biased ways to analyze available information (which are simply less precise than the models for active traders).

**Table 6:** Number of traders by volume and activity in both markets.

	Democratic		Republican	
	Large	Small	Large	Small
Active	295	252	413	275
Inactive	764	2,439	980	2,784

In both markets the majority of traders exclusively traded Yes contracts. Across both markets, just 17% traded only No contracts and an additional 22% traded both No and Yes contracts. The remaining 61% traded only Yes contracts. With so many traders only trading Yes contracts and with the structural benefits of No contracts, it is not surprising that there were more profitable trading opportunities for No contracts than Yes contracts.

## Dominant contracts

### Hypothetical market

**Fig 5:**
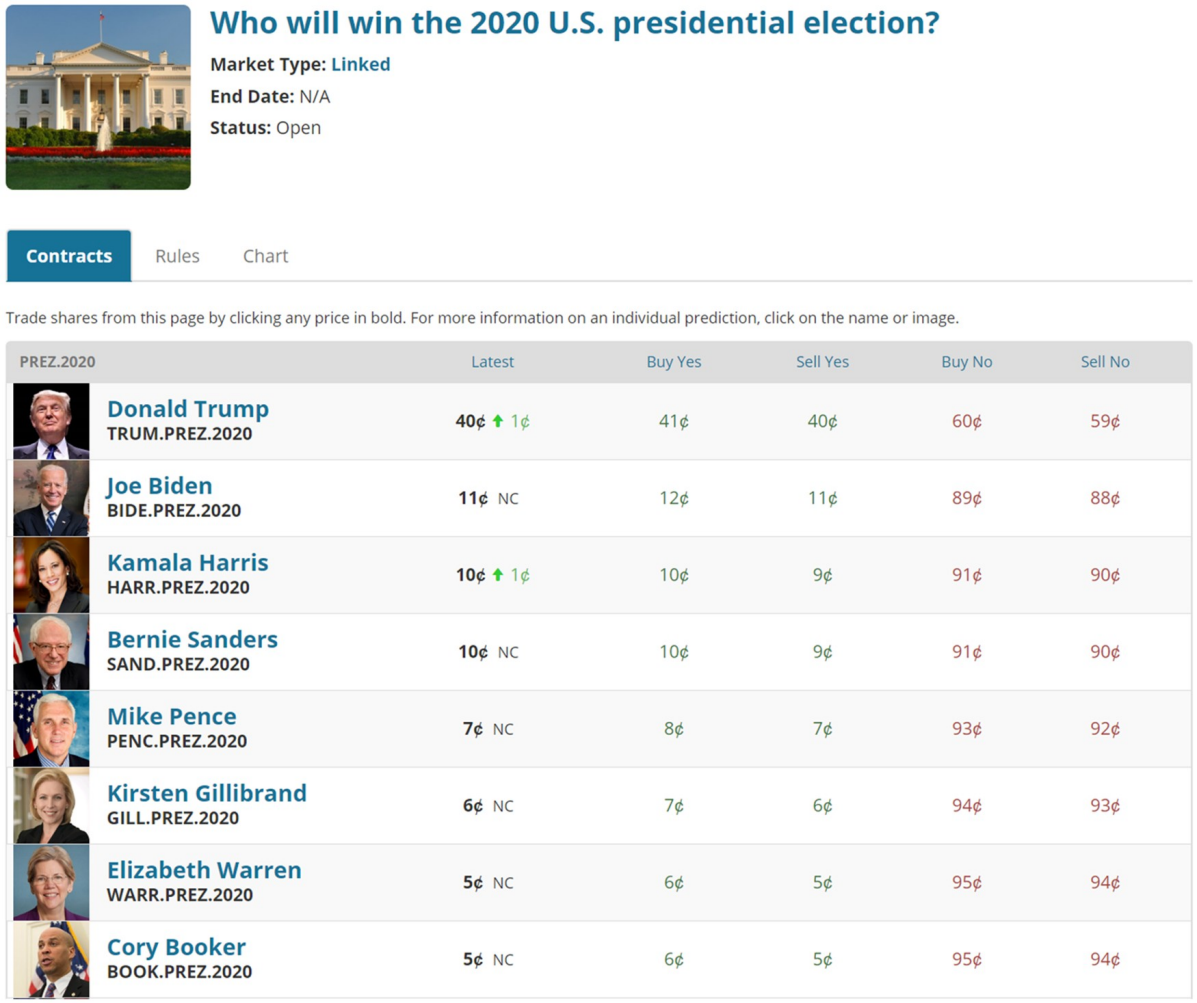
Sample market from PredictIt. Reprinted from PredictIt under a CC BY license, with permission from PredictIt, original copyright 2016.

Consider a hypothetical market within PredictIt with *n* mutually exclusive outcomes. The market provides Yes and No contracts for traders to trade. Each possible outcome has a Yes and No contract with a payoff that is linked to whether or not that outcome is the one that is realized at market termination. A Yes contract is worth $1 if and only if its outcome is realized and $0 otherwise. A No contract is worth $1 if and only if its outcome is not realized and $0 if it is. Before market termination the price of any Yes or No contract is always greater than (or equal to) $0 and less than (or equal to) $1. Fig 5 is image of an actual market in PredictIt where you can see a series of mutually exclusive possible outcomes and a set of Yes and No prices.

We are going to start with two simplifying assumptions that we will later relax: (1) no bid/ask spread and (2) no transaction costs. There are Yes contracts *Y*_*i*_ and No contracts *N*_*i*_ for all *i* in *i* = 1…*n*. Since there is no bid/ask spread, *Y*_*i*_ = 1 − *N*_*i*_ and *N*_*i*_ = 1 − *Y*_*i*_. Since there are no transaction costs, the profit from a Yes contract is 1 − *Y*_*i*_ and the profit from a No contract is 1 − *N*_*i*_. In the real market, PredictIt charges a 10% fee on gains that reduce the profits to (1 − 10%)(1 − *Y*_*i*_) or (1 − 10%)(1 − *N*_*i*_). And as Fig 5 shows, there is a spread between the cost to buy and sell a given contract. Since prices are in penny increments, the spread between the best bid and best ask prices must be a minimum of 1 penny wide.

In this market the sum of the Yes contract prices $\sum_{i=1}^{n}Y_{i}$ may or not be equal to $1.

This market also features margin linking, where the exchange considers the mutual exclusivity of the outcomes and the fact that the maximum possible loss a trader can suffer may be less than the total cost of all of the contracts traded. This possibility is clearly seen when a trader buys multiple No contracts. Because only one outcome *i* = 1..*n* will be realized, only one No contract can lose money; the other No contracts will make money. The amount of money the exchange deducts from each trader’s account is equal to the maximum possible loss the trader can suffer for any outcome given all of the contracts in their possession.

Consider a trader who thinks that the Yes contract linked to outcome *a* is under-priced. The trader can make an investment in 1 share of Yes contract *a* for a price of *Y*_*a*_. The trader’s profit if outcome *a* is realized is 1 − *Y*_*a*_. The trader will suffer a loss of *Y*_*a*_ if outcome *a* is not realized. Alternatively, the trader can replicate the payoff of 1 − *Y*_*a*_ if *a* is realized by trading *c* contracts of every No contract except for the one linked to outcome *a*, where

$$\begin{array}{c} c=\frac{1-Y_{a}}{\sum_{i=1,i\neq a}^{n}1-N_{i}} \end{array}$$ (1)

With this set of No contracts the payoff if *a* is realized is

$$\begin{array}{c} \sum_{i=1,i\neq a}^{n}c\left(1-N_{i}\right) \end{array}$$ (2)

It is easy to show that this is equal to 1 − *Y*_*a*_ if outcome *a* is realized.

Because of margin linking, the trader’s cost for trading this set of No contracts is always equal to the maximum possible loss. The trader’s loss if some other outcome *b* is realized is

$$\begin{array}{c} cN_{b}-\sum_{i=1,i\neq a,b}^{n}c\left(1-N_{i}\right) \end{array}$$ (3)

The No contract for outcome *b* suffers a loss but the others in the traded set earn a profit of 1 − *N*_*i*_ per share. It can be shown that the net result for outcome *b* and all the other outcomes *i* = 1..*n*, *i* ≠ *a* are equal to each other. We can see this by setting two loss equations equal to each other and simplifying (shown in Appendix A).

Now, when is it a better choice to buy this set of No contracts instead of a Yes contract? The optimal choice is the one that can be purchased for the lowest cost but with the same payoff if outcome *a* is realized.

With the first simplifying assumption, we apply the pricing relationship of *Y*_*i*_ = 1 − *N*_*i*_ to convert all of the variables to prices of Yes contracts. Thus, Yes contracts are more expensive for the same return if:

$$\begin{array}{c} Y_{a}>c\left(1-Y_{b}\right)-\sum_{i=1,i\neq a,b}^{n}cY_{i} \end{array}$$ (4)

with

$$\begin{array}{c} c=\frac{1-Y_{a}}{\sum_{i=1,i\neq a}^{n}Y_{i}} \end{array}$$ (5)

Which simplifies to:

$$\begin{array}{c} \sum_{i=1}^{n}Y_{i}>1 \end{array}$$ (6)

Buying a replicating set of No contracts is a better choice when the sum of the Yes contract prices is greater than $1. Our empirical observations of this market in Figs 1 and 2 shows that most of the time this was the case. The impact this had on the trader’s transactions is explored in the next section. As this proof suggests, there were many trades for Yes contracts that were more expensive than an equivalent trade executed with No contracts.

Now consider a trader that makes an investment of 1 share of No contract *a* for a price of *N*_*a*_. The trader’s profit if *a* is not realized is 1 − *N*_*a*_. The trader will suffer a loss of *N*_*a*_ if outcome *a* is realized. Alternatively, the trader can replicate the payoff of 1 − *N*_*a*_ if *a* is not realized by trading *d* contracts of every Yes contract except for the one linked to outcome *a*, where

$$\begin{array}{c} d=\frac{1-N_{a}}{1-\sum_{i=1,i\neq a}^{n}Y_{i}} \end{array}$$ (7)

With this set of Yes contracts the payoff if another outcome *b* is realized is

$$\begin{array}{c} d\left(1-Y_{b}\right)-d\sum_{i=1,i\neq a,b}^{n}Y_{i} \end{array}$$ (8)

This is equal to the payoff 1 − *N*_*a*_.

The trader’s loss if outcome *a* is realized is equal to the sum of the Yes contracts purchased. This is the maximum possible loss.

$$\begin{array}{c} d\sum_{i=1,i\neq a}^{n}Y_{i} \end{array}$$ (9)

The formula for *d* in Eq 7 contains a potential problem when replicating a No contract for outcome *a*. The numerator will be negative or zero when

$$\begin{array}{c} 1\leq \sum_{i=1,i\neq a}^{n}Y_{i} \end{array}$$ (10)

This situation will occur in the trading history because $\sum_{i=1}^{n}Y_{i}$ was frequently larger than $1 and there were some candidates who were unlikely to win, with Yes contract prices that were close to $0. Therefore the sum of Yes contract prices excluding just one contract $\sum_{i=1,i\neq a}^{n}Y_{i}$ was also sometimes greater than or equal to $1. This will cause *d* to be undefined or negative.

If *d* is undefined replicating the payoff of the No contract with other contracts is not possible. If *d* is negative then the formula is directing the trader to buy a negative number of Yes contracts. This can be done by instead buying −*d* No contracts. This actually does work and can correctly replicate the payoff of the No contract. Interestingly, the cost of that replicating set of No contracts will also be negative. This means that when this trading opportunity presents itself it is always prudent of the trader to trade the replicating set of No contracts instead of the original No contract.

Another potential problem with *d* in Eq 7 is if the numerator is close to zero. When that happens |*d*| will become very large and in reality the replicating set of contracts may become untradable given PredictIt’s trading limits on individual contracts. In the dataset this situation occurs only a handful of times but it must be articulated for thoroughness. To manage this issue we constrain valid values of *d* to |*d*| < 10. This is a conservative limit that will help us later when we want to make statements comparing the actual trades that took place and the alternative replicating set of contracts that the trader could have traded instead.

To summarize, if *d* is defined and |*d*| < 10 the payoff of a No contract might be replicated by either a set of Yes contracts or a set of No contracts. When is it a better choice to buy the replicating set of contracts instead of the No contract? As before it depends on which choice has the lowest cost for the same payoff. The No contract is more expensive for the same return if:

$$\begin{array}{c} N_{a}>d\sum_{i=1,i\neq a}^{n}Y_{i} \end{array}$$ (11)

with

$$\begin{array}{c} d=\frac{1-N_{a}}{1-\sum_{i=1,i\neq a}^{n}Y_{i}} \end{array}$$ (12)

This is easily reduced to:

$$\begin{array}{c} 1>\sum_{i=1}^{n}Y_{i} \end{array}$$ (13)

Buying the replicating set of contracts is a better choice when the sum of the Yes contract prices is less than $1 (which makes sense with our previous finding on when it is better to buy No versus Yes contracts). Our empirical observations of this market in Figs 1 and 2 shows that this was rarely the case.

Using this simplified model we evaluate if the Yes contract trades are strictly dominated by a replicating set of No contracts. To test this empirically we use the prevailing midquote at the time of each Yes contract buy trade and compared it to the maximum possible loss of a replicating set of No contracts, priced using the prevailing midquotes of the No contracts. We found that for the trades that took place after margin linking was initiated in the Democratic and Republican caucus markets the replicating set of No contracts dominated the Yes contract trades 96.1% and 99.7% of the time, respectively. This means that the majority of the time the No contracts were the cheaper trade. We limited this to only the trades after margin linking was initiated because the earlier trades are less interesting because of the pricing issues shown in Figs 1 and 2.

**Fig 6:**
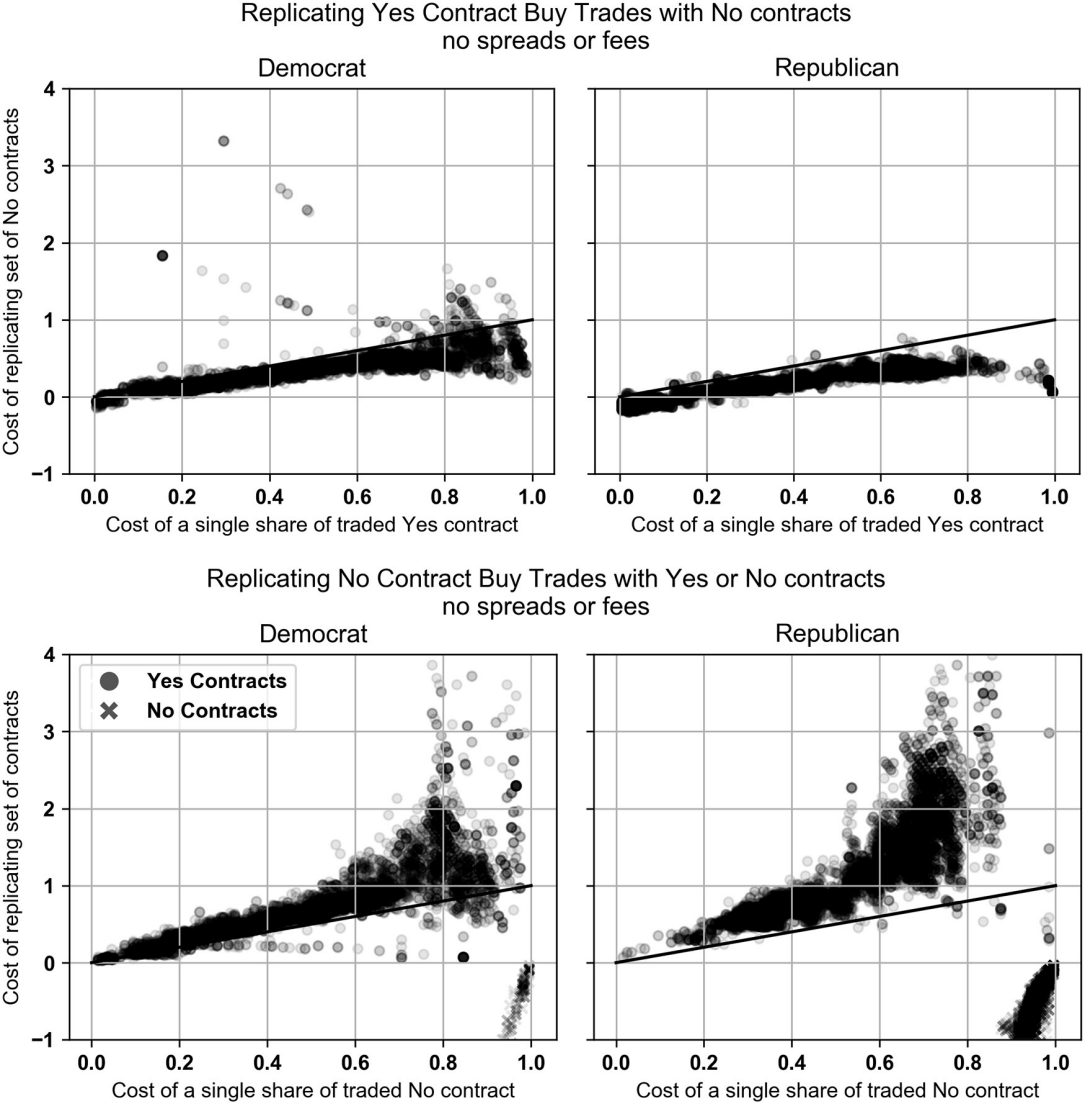
**Top: Buy Yes trades plotted against the optimal replicating set of No contracts. Bottom: Buy No trades plotted against the optimal replicating set of Yes contracts**. All contract trades are priced with the prevailing midquote at the time of the trade. Contract returns do not include PredictIt’s 10% fee. Charts only include trades that took place after margin linking went into effect.

Scatter plots of the data are shown in Fig 6. A black diagonal line is included to clearly show the break-even point where the cost of the Yes contract and the cost of the replicating set of No contracts are equal. Trades that appear below the line are trades that are dominated by the set of No contracts.

We also analyzed the No contract buy trades in both markets and compared them to the replicating set of Yes or No contracts. We found that after margin linking was initiated in the Democratic and Republican Caucus markets, the No contract trades were replicable (meaning *d* is defined and |*d*| < 10) and dominated by the replicating set of Yes contracts only 2.9% and 0.2% of the time, respectively, and by the replicating set of No contracts 1.3% and 41.3%, respectively. These percentages mean that again the majority of the time the No contracts were the cheaper trade. In the Republican market the No contract trades were frequently dominated by a replicating set of No contracts, suggesting that No contracts were still the better option but the traders should have traded different No contracts than the one that actually executed.

Here we are considering only the trades that took place after margin linking was initiated because before margin linking market prices were much more distorted and the results would be less interesting. Additionally, all current PredictIt markets use margin linking, making our results applicable to what a current observer would find in a market today.

### Including market spreads

The equations for *c* and *d* change slightly if the hypothetical market’s prices include bid-ask spreads. If the trader crosses the spread to purchase Yes or No contracts linked to outcome *a*, they are buying contracts at $Y_{a}^{ask}$ or $N_{a}^{ask}$. These trades could each be replicated by crossing the spread and buying a set of No or Yes contracts, respectively. The formulas for *c* and *d* become:

$$\begin{array}{c} c=\frac{1-Y_{a}^{ask}}{\sum_{i=1,i\neq a}^{n}1-N_{i}^{ask}} \end{array}$$ (14)

$$\begin{array}{c} d=\frac{1-N_{a}^{ask}}{1-\sum_{i=1,i\neq a}^{n}Y_{i}^{ask}} \end{array}$$ (15)

If the numerator in Eq 15 is negative then one must instead calculate a replicating set of No contracts with this formula:

$$\begin{array}{c} d^{\prime }=\frac{1-N_{a}^{ask}}{\sum_{i=1,i\neq a}^{n}Y_{i}^{bid}-1} \end{array}$$ (16)

The numerator in Eq 16 may again be negative. If this is the case then there is no replicating set of contracts for that trade.

Our approach becomes more difficult in the case of providing liquidity. If a trader purchases a Yes or No contract by providing liquidity, they had to place an order in an order book and wait for another trader to sell contracts at that price. A trader could also do this for a replicating set of contracts, but it would be uncertain if the trader would be able to execute all of their orders at those prices because the trade is dependent on the arrival of a willing counter-party. For the purpose of our analysis, and our argument of No contracts generally dominating Yes contracts, we will avoid this complexity by instead comparing the price trader’s provide for a trade with the price of the replicating set of contracts if the trader were to instead cross the spread and execute their trades immediately. This is a conservative assumption that results in a higher cost for the trader than the reality of potentially trading at a better price while providing liquidity, but this avoids the uncertainty of whether or not the trader would be able to get filled providing liquidity in the set of contracts at the desired prices.

With this assumption, the formulas for *c* and *d* become:

$$\begin{array}{c} c=\frac{1-Y_{a}^{bid}}{\sum_{i=1,i\neq a}^{n}1-N_{i}^{ask}} \end{array}$$ (17)

$$\begin{array}{c} d=\frac{1-N_{a}^{bid}}{1-\sum_{i=1,i\neq a}^{n}Y_{i}^{ask}} \end{array}$$ (18)

If the numerator in Eq 18 is negative then one must instead calculate a replicating set of No contracts with this formula:

$$\begin{array}{c} d^{\prime }=\frac{1-N_{a}^{bid}}{\sum_{i=1,i\neq a}^{n}Y_{i}^{bid}-1} \end{array}$$ (19)

The numerator in Eq 19 may again be negative. If this is the case then again there is no replicating set of contracts for that trade.

The empirical results changed by a small amount after our analysis included the bid-ask spreads of the contract prices. We analyzed the post-margin linking trades that took liquidity separately from the trades that provided liquidity. For the trades that took liquidity, the Yes contract trades were dominated by the replicating set of No contracts 87.8% and 96.7% of the time in the Democratic and Republican markets, respectively. For the provide trades, the percentages are 66.9% and 94.8%.

Our analysis of the No contract trades continued to show that these trades were rarely dominated by a replicating set of Yes contracts. For the Democratic market, after margin linking went into effect, the No contract trades that took liquidity and provided liquidity were replicated and dominated by a set of Yes contracts 1.7% and 0.9% of the time and by a set of No contracts 1.3% and 0.6% of the time, respectively. For the Republican market, after margin linking went into effect, the No contract trades were replicated and dominated by the set of Yes contracts 0% of the time. The No contract trades were replicated and dominated by the replicating set of No contracts when taking and providing liquidity 39.2% and 24.1% of the time, respectively. Again, the No contracts were still the better option but frequently the traders should have traded different No contracts than the one that actually executed.

### Including market spreads and fees

Now we relax both assumptions and consider the real PredictIt market that has bid-ask spreads and charges a 10% fee on all profits for any trade. Recall that the PredictIt exchange uses a first in, first out accounting model to calculate fees. Fees are based on profitable trading of individual shares, not the net profitability over a day or some other time period. If Trader A sells a contract for a $5 profit, and then loses $15 in the same contract an hour later, they would be charged $0.50 for the profitable trade, even though they later lost $10 that day. Now, because of the fee, the trader’s payoff for any profitable No contract trade is reduced by an amount that is proportional to $1-N_{i}^{ask}$. As a result, the number of shares to buy for each No contract to replicate the payoff of a Yes contract is no longer a constant value *c*. The number of shares of each contract needs to be increased or decreased slightly for the trader to trade optimally. The optimal trade will minimize the maximum possible loss for any No contract, minimizing the amount of money deducted from the trader’s account by PredictIt to cover the margin linking.

**Fig 7:**
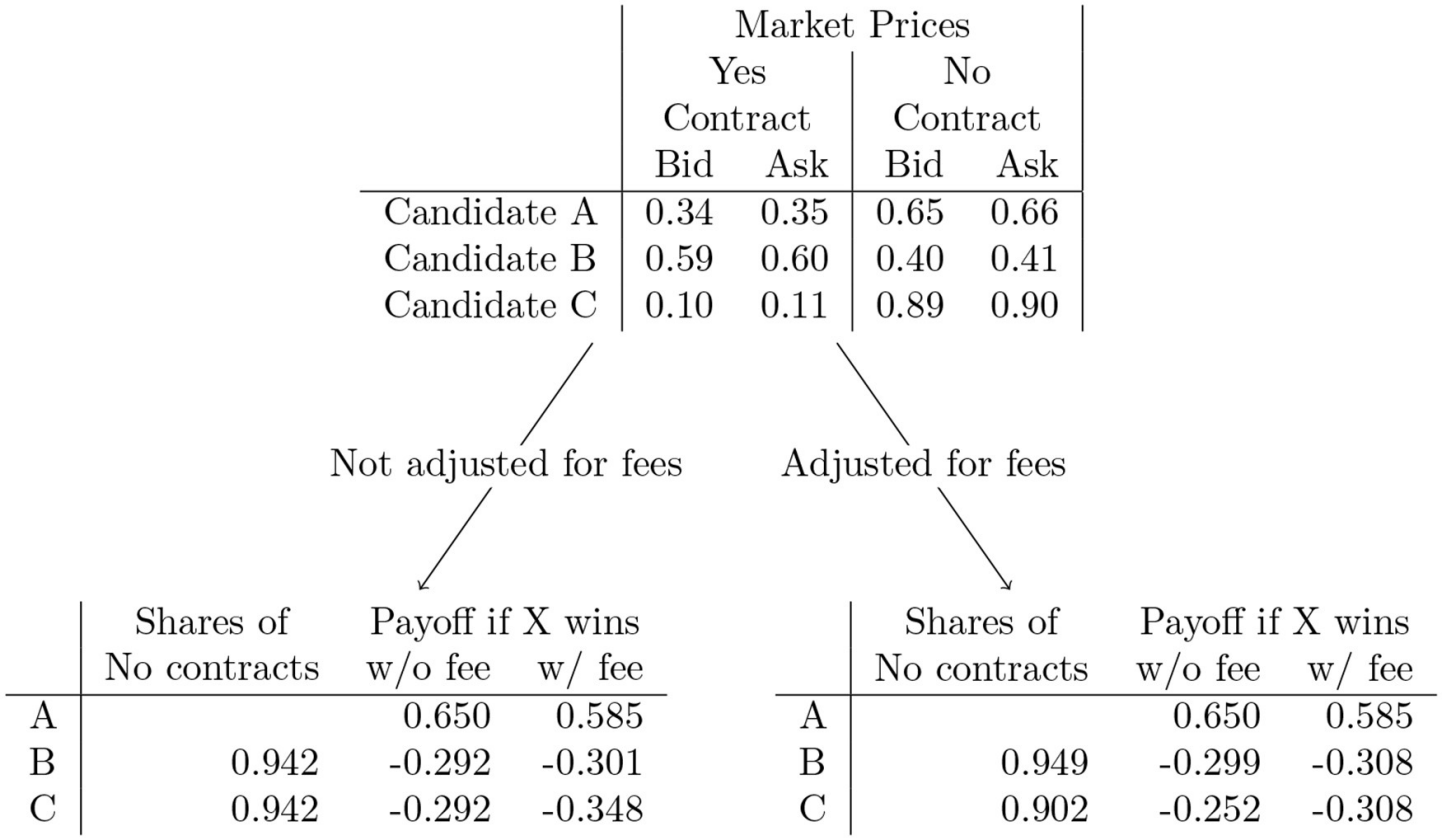
Contract payoffs for a replicating set of No contracts with the number of No contracts adjusted or not adjusted to consider fees. Observe on the left that when the number of No contracts purchased for candidates B and C is the same the loss if candidates B or C win is the same before fees but is different after fees. The number of No contracts purchased can be slightly adjusted to make the losses equal if candidates B or C win, as shown on the right.

It is helpful to illustrate this with a concrete example. Consider a market with contract prices as shown in the top of Fig 7 and a trader that wants to use No contracts to replicate the returns of a single Yes contract for candidate A. If the trader bought 0.942 shares of No contracts for candidates B and C using (14), the net loss if candidates B or C wins will be slightly different from each other because of the fee charged for the one profitable No contract. Because of margin linking, the PredictIt exchange will use the maximum possible loss to determine how much money to deduct from the trader’s account. In this case the amount is 0.348 to cover the loss if candidate C wins. Note that if a fourth candidate, Candidate D, is added to the market and wins the election, the trader will lose money if they had bought Yes contracts for Candidate A. If they had instead bought No contracts for Candidates B and C, they would make a profit.

This isn’t optimal, however. By slightly increasing the number of No contracts for candidate B and decreasing the number of No contracts for candidate C the trader can decrease the maximum possible loss to 0.308, resulting in less money being deducted from their account through margin linking. This is illustrated in the lower right of Fig 7. When considering fees it is necessary to adjust the quantities to achieve the minimum cost replicating set of contracts.

To calculate the formula for the number of shares *c*_*i*_ for each No contract *N*_*i*_, start with the same payoff equation from the previous section but reduce each payoff to account for the fee *f*:

$$\begin{array}{c} \left(1-f\right)\left(1-Y_{a}^{ask}\right)=\left(1-f\right)\sum_{i=1,i\neq a}^{n}c_{i}*\left(1-N_{i}^{ask}\right) \end{array}$$ (20)

Our goal is to find a formula for the number of each No contract to buy, *c*_*i*_, for *i* = 1..*n*, *i* ≠ *a*, to replicate the payoff of Yes contract *Y*_*a*_.

If outcome *a* is not realized then some other outcome *b* has occurred and contract *Y*_*b*_ is worth $1 and *N*_*b*_ is worth $0. The trader’s loss will be:

$$\begin{array}{c} c_{b}N_{b}^{ask}-\left(1-f\right)\sum_{i=1,i\neq a,b}^{n}c_{i}\left(1-N_{i}^{ask}\right) \end{array}$$ (21)

This loss must be equal to the loss of some other potential outcome *x*.

$$\begin{array}{c} c_{b}N_{b}^{ask}-\left(1-f\right)\sum_{i=1,i\neq a,b}^{n}c_{i}\left(1-N_{i}^{ask}\right)=c_{x}N_{x}^{ask}-\left(1-f\right)\sum_{i=1,i\neq a,x}^{n}c_{i}\left(1-N_{i}^{ask}\right) \end{array}$$ (22)

Solving for *c*_*x*_ gives:

$$\begin{array}{c} c_{x}=\frac{\left(1-Y_{a}^{ask}\right)\prod_{k=1,k\neq a,x}^{n}g\left(N_{k}^{ask},f\right)}{\sum_{i=1,j\neq a}^{n}\left(1-N_{i}^{ask}\right)\prod_{w=1,w\neq a,i}^{n}g\left(N_{w}^{ask},f\right)} \end{array}$$ (23)

with

$$\begin{array}{c} g(p,f)=p+(1-f)(1-p)=(1-f)+fp \end{array}$$ (24)

Observe that when the fee *f* → 0, *g*(*p*, *f*)→1 and *c*_*x*_ reduces to 14. Also notice what function *g* represents. This is the amount of money returned to the trader’s account when a No contract purchased for *N*^*ask*^ closes out for $1, net of fees. This amount varies by a small amount based on the value of *N*^*ask*^.

We can also derive a formula for *d*_*x*_ that will enable a trader to replicate the payoff of a single No contract with a set of Yes contracts.

Consider the payoff of a single No contract for outcome *a*. If some other outcome *b* is realized, it will have a positive payoff that will be reduced by the fee *f*. The replicating set of Yes contracts must have the same payoff for outcome *b*. That leads to the equality

$$\begin{array}{c} \left(1-f\right)\left(1-N_{a}^{ask}\right)=\left(1-f\right)d_{b}\left(1-Y_{b}^{ask}\right)-\sum_{i=1,i\neq a,b}^{n}d_{i}Y_{i}^{ask} \end{array}$$ (25)

The payoff of the set of Yes contracts for all outcomes other than *a* must be equal to each other. Therefore, we also know that

$$\begin{array}{c} \left(1-f\right)d_{b}\left(1-Y_{b}^{ask}\right)-\sum_{i=1,i\neq a,b}^{n}d_{i}Y_{i}^{ask}=\left(1-f\right)d_{x}\left(1-Y_{x}^{ask}\right)-\sum_{i=1,i\neq a,x}^{n}d_{i}Y_{i}^{ask} \end{array}$$ (26)

Solving for *d*_*x*_ gives:

$$\begin{array}{c} d_{x}=\frac{\left(1-f\right)\left(1-N_{a}^{ask}\right)\prod_{k=1,k\neq a,x}^{n}g\left(Y_{k}^{ask},f\right)}{\left(1-f\right)\left(1-Y_{x}^{ask}\right)\prod_{k=1,k\neq a,x}^{n}g\left(Y_{k}^{ask},f\right)-\sum_{i=1,i\neq a,x}^{n}Y_{i}^{ask}\prod_{w=1,w\neq a,i}^{n}g\left(Y_{w}^{ask},f\right)} \end{array}$$ (27)

The *d*_*x*_ values may be negative, indicating that the No contract cannot be replicated by Yes contracts. To replicate with other No contracts instead, the following formula can be used:

$$\begin{array}{c} d_{x}^{\prime }=\frac{\left(1-f\right)\left(1-N_{a}^{ask}\right)\prod_{k=1,k\neq a,x}^{n}g\left(N_{k}^{ask},f\right)}{-N_{x}^{ask}\prod_{k=1,k\neq a,x}^{n}g\left(N_{k}^{ask},f\right)+\left(1-f\right)\sum_{i=1,i\neq a,x}^{n}\left(1-N_{i}^{ask}\right)\prod_{w=1,w\neq a,i}^{n}g\left(N_{w}^{ask},f\right)} \end{array}$$ (28)

If the $d_{x}^{\prime }$ values are also negative then the No contract cannot be replicated with either Yes or No contracts.

**Fig 8:**
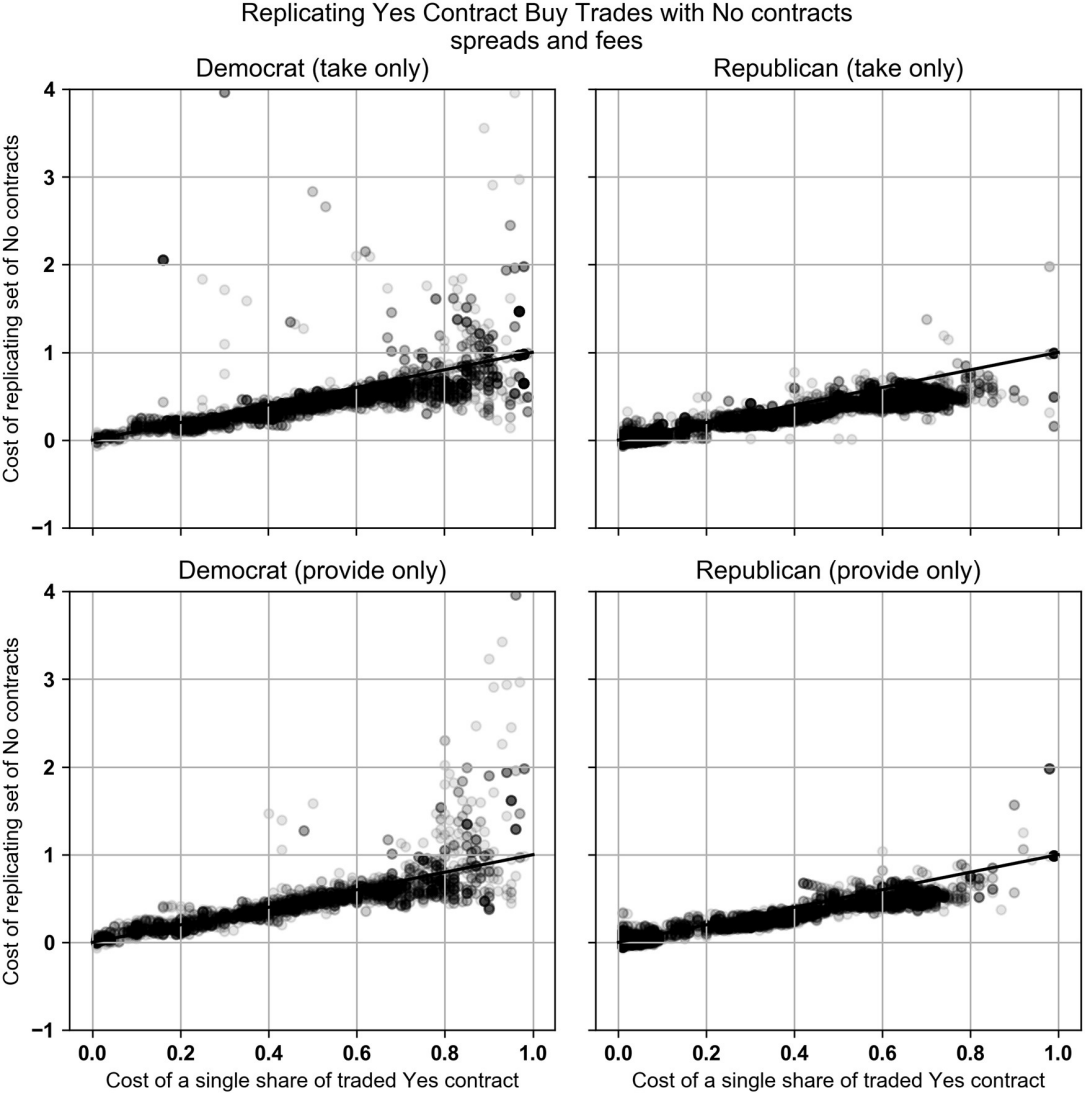
Buy Yes trades that took liquidity plotted against the optimal replicating set of No contracts. All trades are priced using the contract’s bid-ask spread. Contract returns include PredictIt’s 10% fee. Charts only include trades that took place after margin linking went into effect.

Our empirical results changed when our equations considered PredictIt’s 10% fee on each profitable trade. For Democratic market, the Buy Yes trades were replicated and dominated by the replicating set of No contracts 87.6% and 63.9% of the time for the take and provide trades. For the Republican market, the percentages are 89.6% and 83.2%. Scatter plots of the data are available in Fig 8.

**Fig 9:**
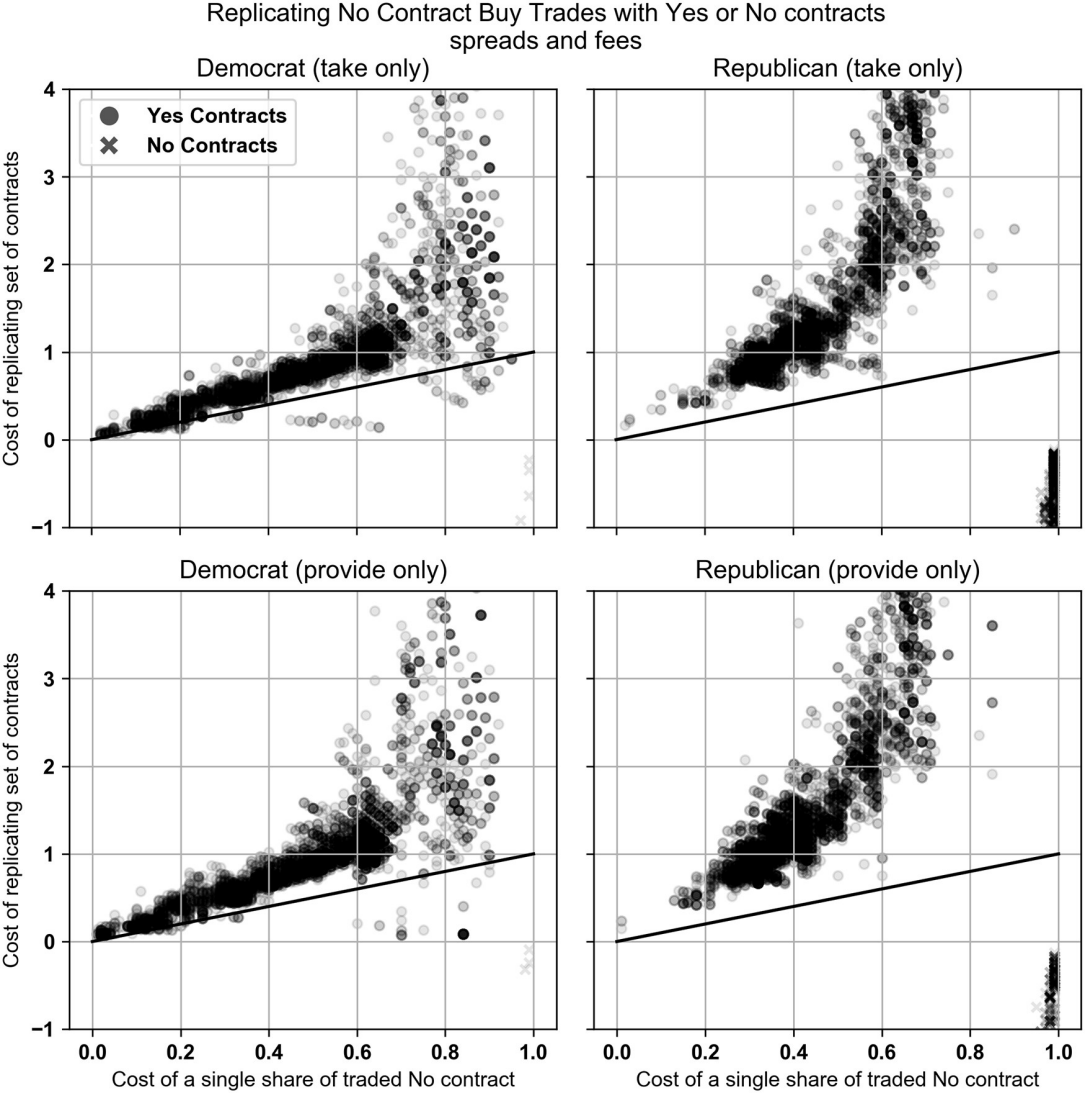
Buy No trades that took liquidity plotted against the optimal replicating set of Yes or No contracts. All trades are priced using the contract’s bid-ask spread. Contract returns include PredictIt’s 10% fee. Charts only include trades that took place after margin linking went into effect.

When comparing the No contract buy trades with the replicating set of Yes contracts, the No contract was rarely dominated by either Yes or No contracts. In the Democratic market the No contract trades were replicated and dominated by a set of Yes contracts 1.7% and 0.9% of the time and by a set of No contracts 0.3% and 0.1% of the time when taking and providing liquidity. In the Republican market, the No contract trades were dominated by a set of Yes contracts 0% of the time. The No contract trades were dominated by a set of No contracts 12.8% and 6.7% of the time when taking and providing liquidity. Charts of the data are shown in Fig 9.

As previously noted, the introduction of margin linking had a large impact on market structure and the majority of trades took place after margin linking went into effect. Still, it is worthwhile to show the equivalent pre-margin linking results for Figs 8 and 9.

While performing this analysis on the pre-margin linking period we were faced with the additional challenge of working with data from before all of the contracts had traded their first share. These trades were considered not replicable and therefore not dominated by a replicating set of contracts.

**Fig 10:**
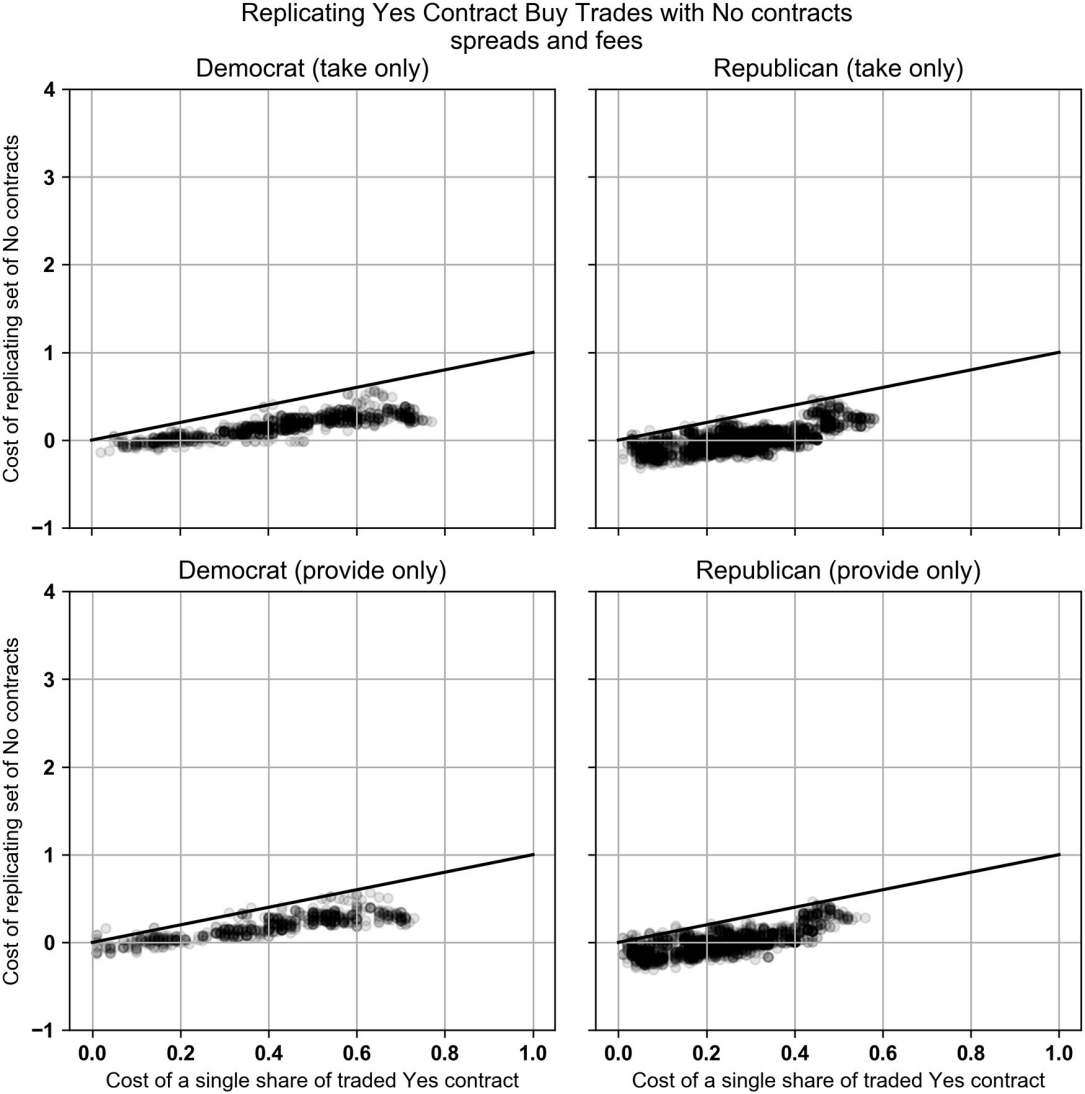
Buy Yes trades that took liquidity plotted against the optimal replicating set of No contracts. All trades are priced using the contract’s bid-ask spread. Contract returns include PredictIt’s 10% fee. Charts only include trades that took place before margin linking went into effect.

For the pre-margin linking period in the Democratic market, the Buy Yes trades were replicated and dominated by a set of No contracts 100% and 98.7% of the time for the take and provide trades. In the Republican market the percentages are 99.7% and 99.1%. See Fig 10 for Scatter plots. These results indicate that trading Yes contracts was almost always inferior to trading No contracts before margin linking began. This is consistent with Figs 1 and 2.

**Fig 11:**
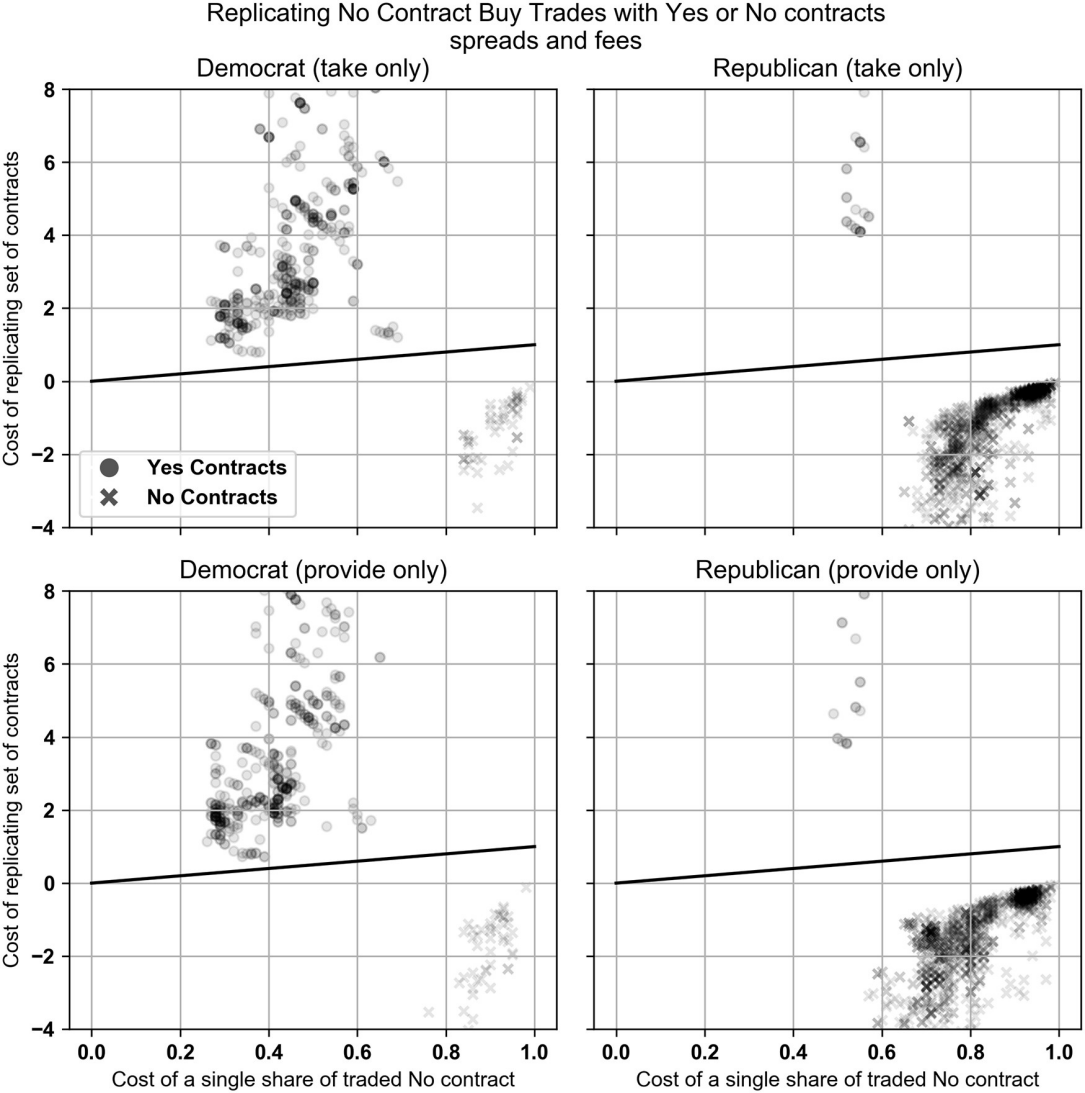
Buy No trades that took liquidity plotted against the optimal replicating set of Yes or No contracts. All trades are priced using the contract’s bid-ask spread. Contract returns include PredictIt’s 10% fee. Charts only include trades that took place before margin linking went into effect.

The Buy No trades were never replicated and dominated by a set of Yes contract trades during the pre-margin linking period for either the Democratic or Republican markets, but in an odd quirk of pre-margin linking, many No contracts were dominated by other sets of No contracts because the prices were so distorted. In the Democratic market the Buy No trades were replicated and dominated by a set of No contracts 14.3% and 15.3% of the time when taking and providing liquidity. In the Republican market, the percentages are 97.9% and 98.6%. Scatter plots of these results are in Fig 11.

### Evaluating trader efficiency

We can employ our concept of dominated contracts to evaluate all of the executed trades, both before and after margin linking was implemented, and determine if they are or are not dominated by a replicating set of contracts. We are including trades executed in the pre-margin linking period because some traders only executed trades during that time and without including those trades we would have no way of classifying those traders. Trades that are replicable and are dominated by the replicating set of contracts are deemed inefficient trades. Trades that are either not replicable or are not dominated by the replicating set of contracts are deemed efficient trades. Note that the way we have defined efficiency, there is no plausible reason for someone to desire the inefficient contract.

We can then look at each traders’ executed volume and determine what percent was executed efficiently. As noted above in the Trader Behavior Summary section, we classify traders that executed at least 75% of their volume efficiently as efficient traders; the remaining are classified as inefficient traders. Our results are robust to changes to the cutoff percentage.

## Results

Using the metrics described in the Trader Behavior Summary and Evaluating Trader Efficiency sections, we categorized all of the traders in each market according to the size, activity, and efficiency metrics, labeling each as small or large, inactive or active, and inefficient or efficient. We organized similar traders into subgroups and analyzed the trading behavior and profitability of each subgroup separately.

We found that the largest group of traders in both markets were inefficient, small, and inactive. Table 7 shows that nearly half of all traders are in this category. Further, about 70% of all traders are inefficient and roughly the same amount of are small, but about 85% are inactive.

**Table 7:** Percentage of traders by volume, activity, and efficiency in both markets. **Profit: net of fees, per trader**. Exposure: max total exposure, per trader. ROI: profit over Exposure, per trader. There were 3,750 traders in the Democratic market and 4,452 in the Republican market.

	% of Traders		Exposure ($)		Profit ($)		ROI	
	D	R	D	R	D	R	D	R
Efficient Small Active	2.21	0.87	19.20	19.78	0.62	4.65	3.27	23.49
Efficient Small Inactive	19.33	16.13	13.16	12.62	-2.12	0.511	-16.15	4.05
Efficient Large Active	3.23	2.07	463.66	519.13	24.87	-1.28	5.36	-0.24
Efficient Large Inactive	7.44	6.22	298.55	338.21	37.16	36.70	12.45	10.85
Inefficient Small Active	4.51	5.30	17.66	18.11	-4.21	0.976	-23.82	5.39
Inefficient Small Inactive	45.71	46.41	12.81	12.94	-4.04	-1.41	-31.52	-10.88
Inefficient Large Active	4.64	7.21	455.57	448.94	9.46	-25.56	2.08	-5.69
Inefficient Large Inactive	12.93	15.79	335.07	309.84	-36.33	-32.45	-10.84	-10.47

Profits are dispersed, but two things are clear: efficient traders were generally profitable, and while there is a mix of profits and losses, there are larger absolute values for groups with higher average exposures (which makes sense, as they are risking more money).

Dividing profits by Exposure provides the Return on Investment or ROI. This helps scale trader profits by the capital risk to better compare the success of the different approaches to trading.

We decompose gross profits into three categories: spread profit, bias profit, and position profit.

The spread profit is the accumulation of small price gains from trading at a better price by providing liquidity instead of taking liquidity. We calculated this as the difference between the executed price and the contract midquote at the time of each execution. Observe that in general the traders who were efficient or active were better at earning spread profits.

The bias profit is the accumulation of gains from the price distortion associated with a market’s set of contract prices adding up to more than $1. To calculate this we first normalized the contract midquote of each executed contract by the sum of all the midquotes. The bias profit was the difference between the normalized midquote and the actual midquote. As expected, efficient traders had positive bias profits.

The position profit is all of the remaining gross profits after spread and bias profits are removed. This is the profit from favorable price movements of the contracts. Traders that were better at identifying the right candidates at the right time were better at accumulating position profits.

PredictIt’s 10% fee is subtracted from the gross profit to yield the net profit. We show how all of our categories did by profit type in Table 8.

**Table 8:** Spread, bias, position, fee are all components of net profit and are divided by exposure, per trader, for comparison purposes. There were 3,750 traders in the Democratic market and 4,452 in the Republican market.

	Net Profit		Spread		Bias		Position		Fee	
	D	R	D	R	D	R	D	R	D	R
Efficient Large Active	5.4	-0.2	7.3	8.1	4.4	5.9	-2.6	-9.7	-3.8	-4.6
Efficient Small Active	3.3	23.5	3.5	0.7	5.0	0.7	-2.1	27.0	-3.1	-4.9
Efficient Large Inactive	12.4	10.9	-0.7	-1.7	2.9	6.7	13.6	9.9	-3.3	-4.0
Efficient Small Inactive	-16.1	4.1	-1.0	-3.9	4.6	1.5	-17.4	10.4	-2.3	-3.9
Inefficient Large Active	2.1	-5.7	-0.2	1.8	-0.9	0.3	5.9	-2.9	-2.7	-4.8
Inefficient Small Active	-23.8	5.4	0.5	-0.3	-3.2	-2.8	-18.9	13.6	-2.2	-5.1
Inefficient Large Inactive	-10.8	-10.5	-1.7	-1.7	-2.1	-3.2	-4.6	-1.7	-2.4	-3.9
Inefficient Small Inactive	-31.5	-10.9	-2.3	-2.9	-5.2	-9.4	-22.0	5.6	-1.9	-4.2

Efficient traders earned bias profits by definition. They are frequently making the most efficient trades, and in markets that frequently have inefficiencies, there are many opportunities for earning bias profits. Generally active traders earned spread profits. The active traders know they could accumulate small profits by providing liquidity instead of taking liquidity. Position profits are much more mixed, even for the efficient traders. But, this is not the easiest view to digest, so we reconstitute Table 8 into Table 9 which illustrates how each category compares to the other categories with just one variable shifted. For instance, on the top row we show how Efficient traders do versus Inefficient traders when they are “Large Active”, “Small Active”, “Large Inactive”, and “Small Inactive”. So, Efficient Large Active have 12.8 and 12.0 percentage points higher ROI for Spread + Bias than Inefficient Large Active. But, Efficient Large Active have 8.5 and 6.8 percentage points lower ROI for Position than Inefficient Large Active. Overall, Efficient Large Active have 3.3 and 5.4 percentage points higher ROI than Inefficient Large Active.

**Table 9:** Differences in ROI holding other two variables constant. There were 3,750 traders in the Democratic market and 4,452 in the Republican market.

[Democratic, Republican] in terms of difference in percent ROI				
Efficient—Inefficient	Large Active	Small Active	Large Inactive	Small Inactive
Spread + Bias	12.8, 12.0	11.2, 4.6	6.0, 9.8	11.1, 9.9
Position	-8.5, -6.8	16.8, 13.4	18.2, 11.6	4.7, 4.8
ROI	3.3, 5.4	27.1, 18.1	23.3, 21.3	15.4, 14.9
Active—Inactive	Efficient Large	Efficient Small	Inefficient Large	Inefficient Small
Spread + Bias	9.5, 9.2	5.0, 3.8	2.7, 7.0	4.9, 9.1
Position	-16.2, -19.6	15.3, 16.6	10.5, -1.2	3.1, 8.1
ROI	-7.1, -11.1	19.4, 19.4	12.9, 4.8	7.7, 16.3
Large—Small	Efficient Active	Efficient Inactive	Inefficient Active	Inefficient Inactive
Spread + Bias	3.2, 12.7	-1.4, 7.3	1.5, 5.2	3.7, 7.3
Position	-0.5, -36.7	31.0, -0.5	24.9, -16.5	17.4, -7.2
ROI	2.1, -23.7	28.6, 6.8	25.9, -11.1	20.7, 0.4

Efficient dominates inefficient conditional on the other two variables. Only in Position ROI for Large Active traders are efficient traders not getting a better return than inefficient traders (and their overall ROI is still higher).

Active does very well against inactive conditional on the other two variables. While active traders are not as impressive versus inactive as efficient versus inefficient, they are consistent. Only in the Efficient Large category are they actually getting a lower ROI. Which means efficient large inactive traders do better than efficient large active traders.

Large has relatively mixed results relative to small conditional on the other two variables. Being a large trader does not tell us much about their return on investment.

We run a regression to test what we see in the tables: what is the return on investment with dummy variables for size, activity, and efficiency. We ran this separately for each market. Simple ordinary least squares: ROI = constant + Efficiency + Size + Activity. Results in Table 10.

**Table 10:** Regression coefficients on ROI. * Significant at 5%, ** Significant at 1%.

	Democratic		Republican	
constant	-0.35**	(0.01)	-0.11**	(0.02)
Efficient	0.14**	(0.02)	0.15**	(0.03)
Large	0.20**	(0.02)	0.01	(0.03)
Active	0.13**	(0.03)	0.10*	(0.04)

Efficiency is the only variable that is significant in both markets. Active is slightly smaller and not fully significant in the Republican market, but also consistent. Size is big and positive in the Democratic market, but near zero and non-significant in the Republican market. It is tempting to try to deconstruct the differences between the two markets, but the biggest distinction is the number of competitive candidates. In future work we hope to replicate this over hundreds of markets and deconstruct the correlation of market-level characteristics and our three variables, but until then we using the two markets as adding power to our findings across various types of markets, rather than a test between them.

There is a high degree of classification overlap for the traders that traded in both markets. We found that there were 2,042 traders that traded in both markets; this is 54% of the traders in the Democratic market and 46% of the traders in the Republican market. For those 2,042 traders, 65.6% of them had the same efficiency classification in both markets. Table 11 shows the cross tabulation of the efficiency classification in both markets. We also found that 80.0% had the same size classification and 75.7% had the same activity level classification.

**Table 11:** Efficiency classification cross tabulation in the Democrat and Republican markets for the 2,042 traders that traded in both markets.

	Republican Classification	
	Efficient	Inefficient
Democrat classification		
Efficient	242	437
Inefficient	265	1,098

An important question we need to answer is if the 65.6% overlap of efficiency classification is statistically significant. If the traders who were classified as efficient knew nothing about trading but were classified as efficient because they randomly made trades that happened to be efficient, their efficient classification would be nothing more than an ex-post evaluation and they would not be more likely to trade efficiently in other markets. We wish to show that this is not the case.

To test the significance we can do a simulation to determine the distribution of random classifications. For the 2,042 overlapping traders, 33% are classified as efficient in the Democratic market and 25% are classified as efficient in the Republican market. Both statistics are the same for all of the traders in their respective markets. If we randomly assign classifications to the traders’ activity in each market in these same proportions, we find that 58.3% of the traders have same classification with a standard deviation of 1.1%. Using this distribution we find that the z-score of our 65.6% measurement is 6.7. Clearly this has statistical significance and our results show that trading efficiency is a real trader characteristic for many people. But, there are some traders who are efficient in one market and not the other. These traders are going to be more profitable where they are efficient, and we have no way of knowing if it is luck that occasionally makes an less-knowledgeable person pick the efficient trade or if it is laziness that an efficient person chooses the less efficient option in a given market.

## Discussion

There are a large number of inefficient trades. The vast majority of trades of Yes contracts could have been done with No contracts. If there were institutional traders or enough retail traders who figured this out, the advantage of trading efficiently would have shrunk, but it did not.

Considering that large traders have more wealth on the line, it is remarkable that they did not get better returns with larger volume. Theory would dictate that these traders are not constrained by liquidity, so they can trade efficiently based on their information. Further, they may even have more information than other traders. But, this is not found in our results.

Considering they objectively apply their information, it is reasonable that active traders did better than inactive traders (most of the time). These traders have better models of the available information, which likely also includes being non-partisan in how they trade.

But, most important: understand how markets work. Efficient traders dominate inefficient traders, holding everything else constant. While we solved for efficiency with a reasonably complex model that would have been costly for any retail trader to employ, simple heuristics would have sufficed. For instance, a trader could simply have traded only No contracts whenever the Yes contracts for a set of mutually exclusive outcomes were priced above $1.

We suggest that exchanges like PredictIt should focus on lowering the barriers to efficiency for retail traders. This could be a wizard on the PredictIt confirmation page which would tell users if there is another strictly dominant trade that they should consider instead of their current trade. Or it execute the more efficient trade for the trader, without even telling the trader. For instance: if it a trader wants to buy Trump for President in 2020 for $0.45, but Harris to not be President in 2020 is selling for $0.40, the exchange could simply buy the Harris contract on the back-end, but display the Trump for President to the trader. The exchange could split the savings with either the exchange, buyer, or seller. If both Harris and Trump lose, the exchange could profit, or again share the winnings with either the buyer or seller.

If the exchange made efficient trades easier or automatic, it would eliminate much of the easy profits of the efficient traders. This transfer of wealth from those unknowledgeable about trading to those who are knowledgeable keeps some people in the market, but gets them spending resources on the marginal zero-sum gains, rather than adding information. In theory, this should force some of these more capable traders into information trading (while some, with lower information, may leave the exchange). This could also allow some traders with a lot of information, but little understanding of trading, to make money and move into the market. In net, the trade-offs could lead to less trading, but should lead to higher information in the market.

Critically, this could be an important testing ground to narrowing the advantages that institutional traders have over retail traders in larger financial exchanges. Many retail investors actually know things about companies or the economy, but do not know how to make the trades that could supply this information into the market. Similar to pushes to create exchanges that limit the ability to front-run, exchanges should consider all changes that ensure that main rewards go to traders with information and the liquidity to trade.

## Supporting information

S1 Fig Timing and volume of trades by traders labeled as efficient and inefficient. Top chart is Democratic market and bottom chart is Republican market. The trading volume is highly correlated between groups as it goes up and down over time. And, the vast majority of trades for all groups occurs over the last few days. Thus, while more markets will allow us to further explore the relationship between efficiency and timing, we are confident that efficiency is not a product of the timing of trades.
(TIF)
