# Supplementary figures and images for: Understanding market functionality and trading success
Source: PLoS One. 2019 Aug 21;14(8):e0219606. doi: 10.1371/journal.pone.0219606 (PMC6703684; doi:10.1371/journal.pone.0219606)

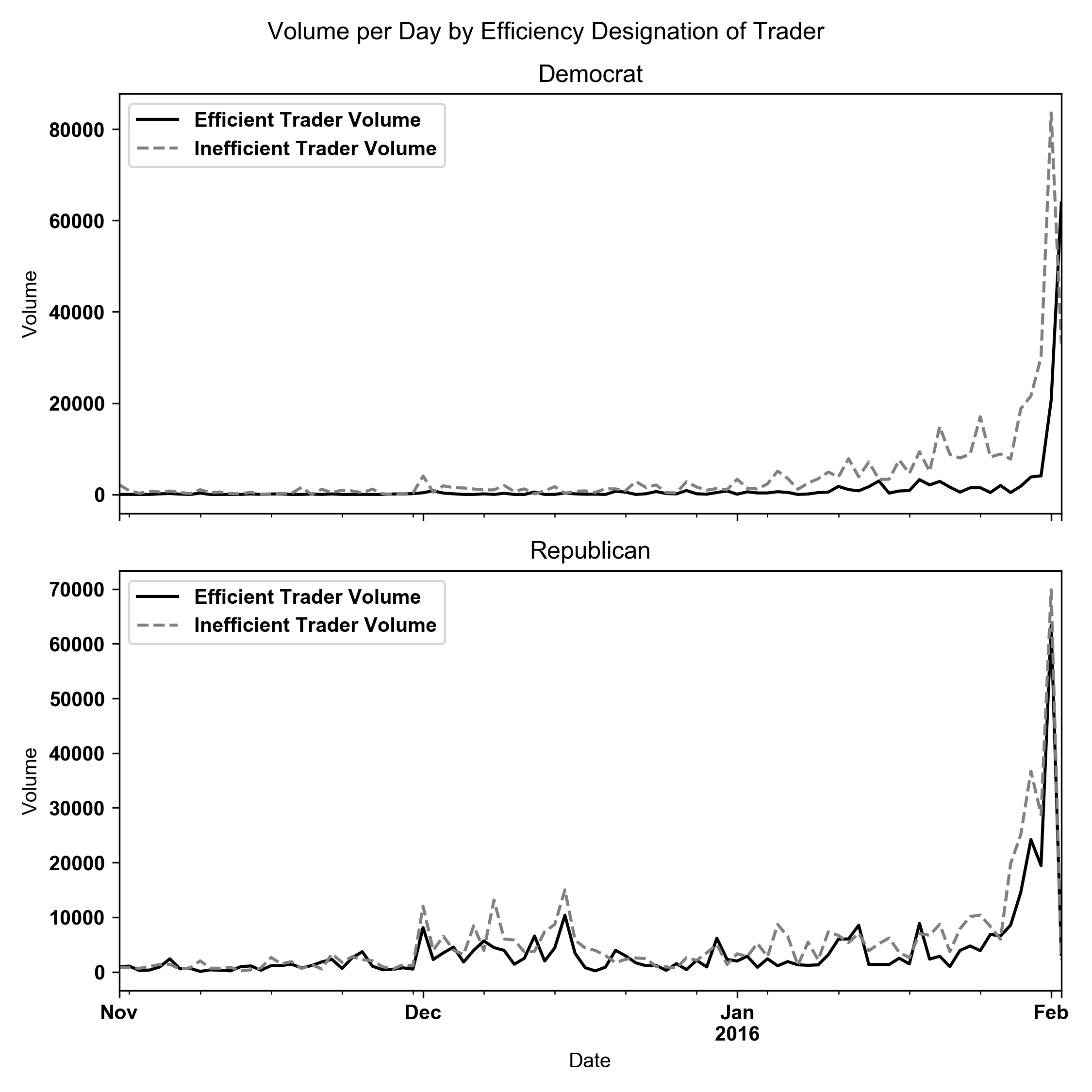

Supplement: S1 Fig — Top chart is Democratic market and bottom chart is Republican market. The trading volume is highly correlated between groups as it goes up and down over time. And, the vast majority of trades for all groups occurs over the last few days. Thus, while more markets will allow us to further explore the relationship between efficiency and timing, we are confident that efficiency is not a product of the timing of trades. (TIF) [file pone.0219606.s001.tif]
